# The longitudinal impact of adverse childhood experiences on college students’ intrinsic motivation for physical exercise: the chain mediating effect of meaning in life and psychological resilience

**DOI:** 10.3389/fpsyg.2026.1875230

**Published:** 2026-07-31

**Authors:** Sicheng Min, Xianxiong Li

**Affiliations:** College of Physical Education, Hunan Normal University, Changsha, China

**Keywords:** adverse childhood experiences, college students, intrinsic motivation for physical exercise, meaning in life, psychological resilience

## Abstract

**Objective:**

This study aimed to examine the longitudinal association between adverse childhood experiences and college students’ intrinsic motivation for physical exercise and to test the individual mediating and chain mediating effects of meaning in life and psychological resilience in this relationship.

**Methods:**

Using convenience sampling, a 6-months longitudinal survey was administered to 667 Hunan college students in Oct 2025 (T1) and Apr 2026 (T2). The measurement instruments included the Adverse Childhood Experiences Questionnaire, the Meaning in Life Questionnaire, the Psychological Resilience Scale, and the Situational Motivation Scale (intrinsic motivation subscale). Mplus 8.0 was used to conduct longitudinal measurement invariance testing, and Model 6 of the PROCESS macro in SPSS 27.0 was employed to perform chain mediation analysis.

**Results:**

T1 adverse childhood experiences significantly negatively predicted T2 intrinsic motivation for physical exercise (β = −0.120, *P* < 0.01). T2 meaning in life played a significant mediating role between T1 adverse childhood experiences and T2 intrinsic motivation for physical exercise, with an indirect effect value of −0.021, accounting for 17.15% of the total effect. T2 psychological resilience also played a significant mediating role, with an indirect effect value of −0.032, accounting for 26.31% of the total effect. The chain mediating effect of T2 meaning in life and T2 psychological resilience was significant, with an effect value of −0.023, accounting for 19.07% of the total effect. The direct effect of T1 adverse childhood experiences on T2 intrinsic motivation for physical exercise was not significant (β = −0.045, *P* > 0.05), indicating that the indirect pathways fully accounted for the total association in this model, with the total indirect effect accounting for 62.53% of the total effect.

**Conclusion:**

Adverse childhood experiences are longitudinally associated with lower levels of intrinsic motivation for physical exercise among college students through the individual mediating pathways of meaning in life and psychological resilience, as well as their chain mediating pathway, with the psychological resilience pathway showing the largest effect size. These findings suggest a pathway through which early adversity may be linked to adult health behavior motivation, providing evidence for integrated physical exercise and mental health interventions in universities.

## 1 Introduction

Regular physical exercise plays a positive role in promoting both physical and mental health (Warburton et al., 2006). Physical exercise not only enhances physical fitness and prevents chronic diseases (Anderson and Durstine, 2019) but also alleviates psychological stress, improves emotional states, and enhances subjective well-being (Mahindru et al., 2023). However, the initiation and maintenance of exercise behavior are not spontaneous processes; rather, they are largely driven by an individual’s internal motivation (Teixeira et al., 2012). Self-Determination Theory posits that intrinsic motivation is an endogenous drive arising from an individual’s interest in and enjoyment of an activity itself, and it plays a critical role in determining the level of participation in and adherence to physical exercise (Ednie and Stibor, 2017; Pedro et al., 2012). Currently, the physical activity situation among Chinese college students is far from optimistic, with over 48% of students engaging in physical activity fewer than 3 times per week (Yu et al., 2025). A lack of intrinsic motivation for physical exercise is a key contributing factor to this situation (Deng et al., 2023; Tao et al., 2019). Therefore, investigating the determinants of college students’ exercise motivation and the mechanisms that influence it is of considerable practical importance for understanding and promoting health behaviors in this population.

Adverse Childhood Experiences (ACEs) refer to potentially traumatic events experienced before the age of 18, including abuse (emotional abuse, physical abuse, and sexual abuse), neglect (emotional neglect and physical neglect), and household challenges (household member substance abuse, parental separation or divorce, household member mental illness, household member criminal behavior, and mother being treated violently) (Hughes et al., 2017). As a prototypical form of early negative environmental exposure, the detrimental consequences of ACEs often manifest in a delayed and covert manner, exerting a persistent and profound impact on an individual’s psychosocial adaptation and behavioral patterns in adulthood (Stonard, 2019; Bevilacqua et al., 2021; Parnes and Schwartz, 2022; Liu et al., 2023). Prior research has demonstrated that adverse health behaviors among college students, including physical inactivity, are positively associated with the severity of their childhood experiences (Krinner et al., 2020; Windle et al., 2018). Therefore, tracing the roots of individual differences in college students’ intrinsic motivation for physical exercise from the perspective of the early life course represents a direction worthy of in-depth investigation.

Although previous studies have identified multiple factors influencing intrinsic motivation for physical exercise, spanning from individual psychology to the social environment, whether and how ACEs shape exercise motivation in adulthood remains an issue that warrants further exploration. To date, few studies have directly examined the relationship between early traumatic experiences and later intrinsic motivation for physical exercise. A deeper analysis of the psychological mechanisms underlying this relationship will help enrich the research perspective on the formation of individual intrinsic motivation for physical exercise. Accordingly, this study proposes meaning in life and psychological resilience as factors in the influencing mechanism and formulates corresponding hypotheses. First, ACEs may impair an individual’s cognitive construction of life goals and values, leading to a decline in meaning in life (Moon and Han, 2022). A deficiency in meaning in life may, in turn, deprive individuals of the intrinsic drive to engage in physical exercise (Sutin et al., 2022). Second, psychological resilience, as an individual’s psychological capacity to cope with stress and achieve positive adaptation, is closely associated with both early adversity and intrinsic motivation for physical exercise (Hu et al., 2025; Shi et al., 2025). Moreover, individuals with a higher sense of meaning in life tend to possess stronger psychological resilience, and psychological resilience may further mediate this influence based on meaning in life (Li D. et al., 2025). In this chain, meaning in life is conceptualized as a more foundational meaning-making system that provides individuals with a cognitive framework for understanding the world and forming life goals, whereas psychological resilience represents a more behaviorally proximal capacity for coping with specific challenges. This conceptual ordering is consistent with the resilience resource model, which posits that a stable value and belief system serves as a prerequisite for effective coping. Taken together, ACEs may not directly undermine college students’ intrinsic motivation for physical exercise; rather, they may exert an indirect effect through the chain psychological pathway of “meaning in life → psychological resilience.” While these theoretical links are plausible, they have rarely been tested simultaneously in a non-Western context, particularly among Chinese college students who navigate a unique educational environment characterized by high academic pressure and specific cultural values regarding physical activity. Therefore, testing this chain mediation model within this specific cultural and educational context is of particular value. This study aims to test this chain mediating model among Chinese college students using a longitudinal tracking design. The findings will provide both a theoretical foundation and empirical support for integrated interventions that combine physical exercise and mental health education in university settings.

### 1.1 The longitudinal association between adverse childhood experiences and college students’ intrinsic motivation for physical exercise

Self-Determination Theory posits that an individual’s intrinsic motivation develops when three basic psychological needs are fulfilled: autonomy, competence, and relatedness. When the environment consistently nurtures these three needs, intrinsic motivation is more likely to develop stably (Ryan and Deci, 2000). Multiple empirical studies have demonstrated that supportive behaviors from family can effectively facilitate adolescents’ internalization of external requirements for physical exercise into voluntary intentions by satisfying these psychological needs, thereby enhancing their exercise participation and adherence (Liu et al., 2025a; Rodrigo et al., 2024). Conversely, the Cumulative Risk Model emphasizes that the greater the number of adversities an individual encounters, the higher the probability of adverse developmental outcomes (Evans et al., 2013; Hardi et al., 2025). ACEs represent a prototypical set of cumulative adversity factors; their presence can severely undermine the safe and supportive environment essential for healthy child development, thereby posing a fundamental threat to the fulfillment of basic psychological needs (Borja et al., 2019). Experiences of abuse may weaken an individual’s sense of autonomy and safety, while experiences of neglect may impair the development of relatedness and competence (Strathearn et al., 2020). Based on this reasoning, early adverse events, acting as cumulative risks, may chronically constrain the normal fulfillment of individuals’ needs for autonomy, competence, and relatedness, potentially setting the stage for a deficiency in intrinsic motivation for physical exercise in adulthood (Grummitt et al., 2021). Based on the above analysis, this study proposes Hypothesis 1 (H1): Adverse childhood experiences will negatively predict the level of intrinsic motivation for physical exercise among college students.

### 1.2 The mediating role of meaning in life

Meaning in life refers to an individual’s overall perception and experience of their purpose in existence, values, and life direction (Boreham and Schutte, 2023). Empirical research has demonstrated that a high level of meaning in life is closely associated with better mental health, higher life satisfaction, and stronger social adaptability (Czekierda et al., 2017; Glaw et al., 2017; Zhang et al., 2024). Moreover, meaning in life provides individuals with intrinsic psychological resources for coping with life challenges (Ward et al., 2023). However, ACEs may fundamentally erode the psychological foundation upon which individuals construct meaning in life (Rose et al., 2023). Relevant studies have found that early experiences of abuse and neglect are often accompanied by low self-worth, impaired interpersonal trust, and negative future expectations, and these maladaptive cognitive tendencies can substantially diminish an individual’s perception of meaning in life (May et al., 2022; Yun et al., 2025). Meanwhile, meaning in life has also been identified as an important factor driving physical exercise behavior (Rush et al., 2021). Longitudinal research has shown that individuals with higher levels of meaning in life during adolescence tend to engage in more frequent physical exercise in subsequent developmental stages (Yemiscigil and Vlaev, 2021). Meaning in life provides individuals with long-term goals and motivation; when individuals perceive their lives as having a clear direction and value, physical exercise is more likely to be regarded as an autonomous activity that is closely connected to self-actualization and imbued with a sense of enjoyment, thereby generating stronger intrinsic drive (Hooker et al., 2024; Kekäläinen et al., 2024). Individuals with a high sense of meaning in life tend to experience more positive emotions and a stronger sense of purpose, which inclines them to engage in proactive reflection, formulate goals and values, and, in turn, incorporate physical exercise as an important pathway for self-actualization (Guo et al., 2024). Taken together, ACEs may first exert a negative influence on individuals’ meaning in life, and this decline in meaning may subsequently impact their intrinsic motivation for physical exercise. Accordingly, this study proposes Hypothesis 2 (H2): Meaning in life plays a significant mediating role between adverse childhood experiences and college students’ intrinsic motivation to engage in physical exercise.

### 1.3 The mediating role of psychological resilience

Psychological resilience refers to an individual’s psychological trait and dynamic capacity to cope effectively and maintain positive adaptation when facing significant stress or adversity (Masten et al., 2021; Sisto et al., 2019). As a crucial psychological resource, psychological resilience plays an important buffering and transformative role between risk factors and individual developmental outcomes (Denckla et al., 2020; Ungar and Theron, 2020). In the context of physical exercise, psychological resilience also plays a role that should not be overlooked. Research has demonstrated that highly resilient individuals are more likely to maintain a positive mindset and sustained willingness when confronting the challenges of physical activity, and their level of intrinsic motivation for exercise participation tends to be higher (Hu et al., 2025; Li N. et al., 2024; Szczegielniak and Krivošová, 2025). Psychological resilience may indirectly facilitate the formation and maintenance of intrinsic motivation for physical exercise through pathways such as enhancing individuals’ self-efficacy, increasing positive emotional experiences, and improving stress-coping styles (Cui et al., 2025; Li X. et al., 2024). Specifically, individuals with high psychological resilience are more adept at adopting positive coping strategies; they can effectively overcome inertia and frustration during exercise and recover rapidly from exercise fatigue or external distractions (Liu et al., 2024; Zhang, 2025). This capacity enables them to maintain stable intentions for participation (Qiu et al., 2025). In contrast, individuals with lower levels of psychological resilience may be more prone to give up when encountering difficulties, fatigue, or setbacks in the exercise process (Li M. et al., 2025). Likewise, existing research has found that individuals who have experienced more adverse events during childhood exhibit lower psychological resilience (Morgan et al., 2021). Based on this evidence, within the longitudinal association between early adversity and exercise motivation, ACEs may reduce individuals’ intrinsic drive to engage in physical exercise in adulthood by weakening the subsequent development of psychological resilience. Accordingly, this study proposes Hypothesis 3 (H3): Psychological resilience mediates the relationship between adverse childhood experiences and college students’ intrinsic motivation for physical exercise.

### 1.4 The chain mediating role of meaning in life and psychological resilience

This study further proposes that a chain mediating relationship may exist between meaning in life and psychological resilience. As an individual’s fundamental cognitive awareness of the purpose and value of their existence, meaning in life often constitutes a more foundational layer of psychological resources. When individuals can clearly perceive direction and meaning in their lives, their psychological resilience in the face of adversity is more readily enhanced (Lasota and Mróz, 2021). One study found that meaning in life serves as an important protective factor for psychological resilience; individuals with a high sense of meaning can discover positive significance amidst difficulties, mobilize internal protective factors, and employ positive emotions to recover from negative affective states, thereby demonstrating stronger psychological resilience (Schaefer et al., 2013). Other empirical research has also confirmed that meaning in life can significantly positively predict psychological resilience (Ostafin and Proulx, 2020). In brief, meaning in life may provide intrinsic belief support and a source of motivation for the development of psychological resilience. Conversely, if individuals fall into a state of meaning deficiency due to early traumatic experiences, their capacity to draw upon internal and external resources to cope with stress may be correspondingly weakened, manifesting as a decline in psychological resilience (Ward et al., 2023). Within the framework of intrinsic motivation for physical exercise formation, ACEs may first erode individuals’ meaning in life; this decline in meaning may subsequently lead to a weakening of psychological resilience, and insufficient psychological resilience may ultimately be reflected in diminished intrinsic motivation for physical exercise (Bertele et al., 2022; Nurius et al., 2015). This mechanism offers a more comprehensive account of how early adversity may profoundly influence adult health behaviors by progressively depleting individuals’ positive psychological capital. Taken together, this study proposes Hypothesis 4 (H4): Meaning in life and psychological resilience chain-mediate the relationship between adverse childhood experiences and college students’ intrinsic motivation for physical exercise.

## 2 Materials and methods

### 2.1 Participants and procedure

Using a convenience sampling method, college student participants were recruited from two universities in Changsha and Xiangtan, Hunan Province, in October 2025 (T1) and April 2026 (T2). Hunan Province, located in central China, bridges the eastern coastal regions and the western inland areas, with a balanced representation of economic development and educational resources, making it a relatively representative region for sampling college students in China. Questionnaire surveys were administered at the two time points, with a 6-months interval between them. The 6-months interval from October to April captures a complete academic semester cycle, including the winter vacation period, during which college students experience a range of meaningful academic, social, and lifestyle transitions that may be associated with changes in psychological resources and exercise behavior. This timeframe is sufficiently long to capture dynamic changes in psychological variables such as meaning in life and resilience, while remaining short enough to minimize participant attrition and ensure data quality (Faden et al., 2004). The survey was administered by uniformly trained class advisors during evening self-study sessions. Data were collected via the Wenjuanxing online survey platform. Prior to the administration, the class advisors provided all participants with a detailed explanation of the research purpose, instructions for completing the questionnaire, and the principles of data confidentiality. After signing an informed consent form, participants voluntarily and anonymously completed the survey and were free to withdraw at any time. The duration of a single session was approximately 20 min.

At T1, a total of 828 questionnaires were collected. After excluding invalid responses (e.g., patterned responding, failure on trap questions, completion time <1 min or >30 min), 753 valid questionnaires remained (valid response rate = 90.94%). At T2, 791 questionnaires were collected from the same participant pool, yielding 703 valid responses after the same exclusion criteria (valid response rate = 88.87%). Matching the two waves of data by student ID numbers resulted in a final valid sample of 667 participants who completed both waves. Due to the anonymous nature of data collection and confidentiality requirements, identifying information of participants who completed only one wave could not be retained. Among the final sample, 342 were male (51.3%) and 325 were female (48.7%). This study was approved by the Ethics Committee of Hunan Normal University (Approval No.: 2025-824), and all research procedures were conducted in accordance with the ethical principles of the Declaration of Helsinki. All participants provided voluntary, informed consent.

### 2.2 Measures

#### 2.2.1 Adverse childhood experiences

The Chinese version of the Adverse Childhood Experiences Questionnaire, adapted and revised by Zhou et al. (2022), was adopted in this study. This instrument encompasses 14 types of adversity, including emotional abuse, physical abuse, sexual abuse, emotional neglect, physical neglect, witnessing violence, witnessing disaster casualties, exclusion/isolation/bullying, family member incarceration, family member mental illness, family member substance abuse, left-behind experience, parental separation/divorce, and loss of a parent. Retrospective measurement was conducted through 22 specific descriptive items, for example: “You were scolded, cursed, insulted, or humiliated by your parents, guardians, or other family members in your home,” “You experienced peer bullying, exclusion, or isolation,” and “Your family was covered by urban or rural subsistence allowances, poverty alleviation programs, or experienced severe financial hardship.” The questionnaire employs a dichotomous scoring format, with each adversity category containing several detailed descriptive items. If any item within a given adversity category is endorsed with a “yes,” that category receives a score of 1, indicating the presence of that adversity. If none of the situations described within a category occurred, a score of 0 is assigned, indicating the absence of that adversity. The scores for all 14 adversity categories are then summed to produce a total ACE score, ranging from 0 to 14, with higher scores indicating a greater magnitude of childhood adverse experiences. In this study, the Cronbach’s α coefficient for this scale was 0.709.

#### 2.2.2 Meaning in life

The Meaning in Life Questionnaire, originally developed by Steger et al. (2006), was adopted in this study. This scale comprises 10 items across two dimensions: presence of meaning and search for meaning. Sample items include: “I am looking for something that makes my life feel meaningful” and “My life has no clear purpose.” The scale employs a 7-point Likert response format, ranging from 1 (absolutely untrue) to 7 (absolutely true), with higher scores indicating a stronger sense of meaning in life. This instrument has been widely utilized among Chinese populations (Jiang and Cao, 2025; Ma et al., 2025). In this study, the Cronbach’s α coefficient for the scale was 0.849 at T1 and 0.952 at T2.

#### 2.2.3 Psychological resilience

The Connor-Davidson Resilience Scale, originally developed by Connor and Davidson (2003), was adopted in this study. This scale comprises 25 items across three dimensions: tenacity, strength, and optimism. Sample items include: “I am able to adapt to change,” “I have close and secure relationships,” and “Past successes give me confidence in facing new challenges.” The scale employs a 5-point Likert response format, ranging from 0 (not true at all) to 4 (true nearly all the time), with total scores ranging from 0 to 100. Higher scores indicate greater psychological resilience. This instrument has been widely utilized among Chinese populations (Dong et al., 2021). In this study, the Cronbach’s α coefficient for the scale was 0.952 at T1 and 0.977 at T2.

#### 2.2.4 Intrinsic motivation for physical exercise

The intrinsic motivation subscale of the Situational Motivation Scale, originally developed by Guay et al. (2000), was adopted as the instrument for measuring college students’ intrinsic motivation for physical exercise in this study. This subscale consists of four items, such as: “Because I think physical exercise is interesting” and “Because physical exercise is fun in itself.” The scale employs a 5-point Likert response format, ranging from 1 (does not correspond at all) to 5 (corresponds exactly), with higher scores indicating a higher level of intrinsic motivation for physical exercise. The validity of this scale has been demonstrated by Clancy et al. (2017). In this study, the Cronbach’s α coefficient for this subscale was 0.932 at T1 and 0.966 at T2.

### 2.3 Data analysis

Data analyses for this study were primarily conducted using SPSS 27.0 and Mplus 8.0 software. The specific steps were as follows. First, Harman’s single-factor test was used to examine common-method bias. Principal component analysis was applied to conduct an exploratory factor analysis of all items. Second, Pearson correlation analysis was used to examine the bivariate associations among the core variables, including T1 ACEs, T1 and T2 meaning in life, T1 and T2 psychological resilience, and T1 and T2 intrinsic motivation for physical exercise. This provided preliminary evidence regarding variable relationships for subsequent mediation model testing. Third, Mplus 8.0 was used to conduct longitudinal measurement invariance testing for meaning in life, psychological resilience, and intrinsic motivation for physical exercise. This process sequentially examined configural, metric, and scalar invariance models. Model fit was evaluated using Chi-square (χ^2^), degrees of freedom (df), Comparative Fit Index (CFI), Tucker-Lewis Index (TLI), Standardized Root Mean Square Residual (SRMR), and Root Mean Square Error of Approximation (RMSEA). Invariance was accepted if changes in fit indices between nested models met established criteria (ΔCFI ≤ 0.015, ΔRMSEA ≤ 0.01). Fourth, Model 6 of the PROCESS macro in SPSS 27.0 was employed to conduct a chain mediation analysis, focusing on testing the longitudinal pathways from T1 ACEs to T2 physical exercise motivation via T2 meaning in life and T2 psychological resilience. The significance of indirect effects was assessed using the bias-corrected bootstrap method with 5,000 resampling iterations. If the 95% confidence interval for a given path did not include zero, the mediating effect was considered statistically significant at the 0.05 level.

## 3 Results

### 3.1 Common method bias test

The Harman single-factor test was applied to the collected data to assess common method bias. An exploratory factor analysis was conducted on all items from the scales measuring T1 ACEs, T1 and T2 meaning in life, T1 and T2 psychological resilience, and T1 and T2 intrinsic motivation for physical exercise. The results revealed that 20 factors with eigenvalues greater than 1 were extracted, and the first factor accounted for 27.76% of the total variance, which is below the critical threshold of 40%. Thus, no serious common method bias was present in this study.

### 3.2 Measurement invariance analysis

Prior to testing the main hypotheses, longitudinal measurement invariance across the two time points was examined for the three scales–meaning in life, psychological resilience, and intrinsic motivation for physical exercise–using Mplus 8.0. The purpose of this analysis was to evaluate whether the measurement structure and meaning of each scale remained equivalent between T1 and T2. The testing procedure sequentially assessed the configural invariance model (M1), the metric invariance model (M2), and the scalar invariance model (M3). The model fit indices and model comparison results are presented in detail in Table 1. As shown in Table 1, the scalar-invariance models for all three scales provided a good fit. The changes in the comparative fit index (CFI) and the root mean square error of approximation (RMSEA) between nested models were minimal (ΔCFI ≤ 0.015, ΔRMSEA ≤ 0.01), fully meeting the established criteria for accepting measurement invariance. These results confirm that the factor structures, factor loadings, and item intercepts of the three scales remained stable across the 6-months follow-up period. This provides robust support for the validity of the subsequent longitudinal data analyses.

**TABLE 1:** Testing for longitudinal invariance of data from three surveys.

Variable	Model	X^2^	df	CFI	TLI	SRMR	RMSEA	Model comparison	ΔCFI	ΔRMSEA
MIL	M1	415.28	159	0.934	0.921	0.052	0.049			
	M2	433.17	168	0.932	0.922	0.054	0.048	M2-M1	−0.002	−0.001
	M3	462.91	178	0.927	0.921	0.055	0.055	M3-M2	−0.005	0.001
PR	M1	1784.32	1149	0.938	0.931	0.045	0.049			
	M2	1828.67	1173	0.936	0.931	0.047	0.049	M2-M1	−0.002	0.000
	M3	1901.48	1198	0.932	0.928	0.048	0.051	M3-M2	−0.004	0.002
IMFPE	M1	74.664	15	0.965	0.934	0.015	0.077			
	M2	79.821	18	0.963	0.943	0.023	0.072	M2-M1	−0.002	−0.005
	M3	87.024	21	0.961	0.948	0.02	0.069	M3-M2	−0.002	−0.003

MIL, meaning in life; PR, psychological resilience; IMFPE, intrinsic motivation for physical exercise.

### 3.3 Descriptive statistics and correlation analysis

A prerequisite for testing mediation effects is the existence of correlations among the variables. The Pearson correlation coefficients among the variables are presented in Table 2. T1 ACEs were significantly negatively correlated with T1 meaning in life, T2 meaning in life, T1 psychological resilience, T2 psychological resilience, T1 intrinsic motivation for physical exercise, and T2 intrinsic motivation for physical exercise. In addition, T1 meaning in life, T2 meaning in life, T1 psychological resilience, T2 psychological resilience, T1 exercise motivation, and T2 intrinsic motivation for physical exercise were all significantly positively correlated with one another.

**TABLE 2:** Descriptive statistics and correlations of study variables (*N* = 667).

Variables	1	2	3	4	5	6	7
T1 ACEs	–	–	–	–	–	–	–
2. T1 MIL	−0.107**	–	–	–	–	–	–
3. T2 MIL	−0.121**	0.296***	–	–	–	–	–
4. T1 PR	−0.179***	0.531***	0.365***	–	–	–	–
5. T2 PR	−0.154***	0.312***	0.546***	0.537***	–	–	–
6. T1 IMFPE	−0.180***	0.334***	0.276***	0.513***	0.386***	–	–
7. T2 IMFPE	−0.120**	0.168***	0.369***	0.240***	0.453***	0.367***	–
M	2.61	49.16	48.84	82.24	85.81	15.37	15.22
SD	2.069	9.117	11.408	16.075	19.299	3.602	3.950

ACEs, adverse childhood experiences; MIL, meaning in life; PR, psychological resilience; IMFPE, intrinsic motivation for physical exercise. ***P < 0.001; **P < 0.01.

### 3.4 Chain mediating effect test of meaning in life and psychological resilience

**FIGURE 1:**
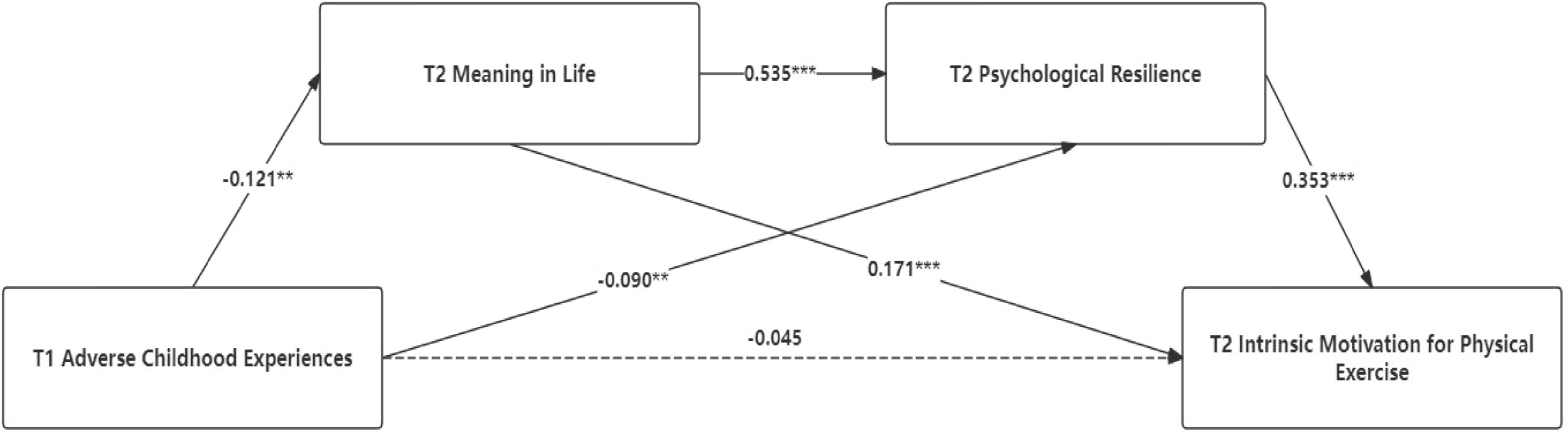
Model diagram of chain intermediary. ***P < 0.001; **P < 0.01.

With T1 ACEs serving as the independent variable, T2 intrinsic motivation for physical exercise as the dependent variable, and T2 meaning in life and T2 psychological resilience as the mediating variables, Model 6 of the PROCESS macro in SPSS was employed to test the chain mediation effect (see Figure 1 and Table 3). In Model 1, which included no mediating variables, the total effect of T1 ACEs on T2 intrinsic motivation for physical exercise was significant (β = −0.120, *P* < 0.01). The results of Model 2 showed that T1 ACEs significantly negatively predicted T2 meaning in life (β = −0.121, *P* < 0.01). The results of Model 3 showed that T1 ACEs significantly negatively predicted T2 psychological resilience (β = −0.090, *P* < 0.01), while T2 meaning in life significantly positively predicted T2 psychological resilience (β = 0.535, *P* < 0.001). The results of Model 4 indicated that after simultaneously entering T1 ACEs, T2 meaning in life, and T2 psychological resilience, the direct effect of T1 ACEs on T2 intrinsic motivation for physical exercise was not significant (β = −0.045, *P* > 0.05), whereas T2 meaning in life (β = 0.171, *P* < 0.001) and T2 psychological resilience (β = 0.353, *P* < 0.001) both had significant positive predictive effects on T2 intrinsic motivation for physical exercise. According to the regression path coefficients described above, T1 ACEs could indirectly predict T2 intrinsic motivation for physical exercise through three distinct pathways: the single mediation pathway of T2 meaning in life, the single mediation pathway of T2 psychological resilience, and the chain mediation pathway of “T2 meaning in life → T2 psychological resilience.” The non-significant direct effect of T1 ACEs on T2 intrinsic motivation for physical exercise in Model 4 indicates that the indirect pathways fully accounted for the total association in this specific model, rather than implying complete mediation in a broader causal sense.

**TABLE 3:** Test for the chain mediation model.

Model	Regression equation		Overall fit indices			Significance		
	Outcome variables	Predictive variables	*R*	*R* ^2^	*F*	β	*t*	95% CI
1	T2 IMFPE	T1 ACEs	0.120	0.014	9.737	−0.120	−3.120**	[−0.196, −0.045]
2	T2 MIL	T1 ACEs	0.121	0.015	9.884	−0.121	−3.144**	[−0.197, −0.045]
3	T2 PR	T1 ACEs	0.553	0.306	146.499	−0.090	−2.750**	[−0.154, −0.026]
		T2 MIL				0.535	16.438***	[0.471, 0.599]
4	T2 IMFPE	T1 ACEs	0.478	0.228	65.331	−0.045	−1.302	[−0.113, 0.023]
		T2 MIL				0.171	4.185***	[0.091, 0.251]
		T2 PR				0.353	8.614***	[0.272, 0.433]

ACEs, adverse childhood experiences; MIL, meaning in life; PR, psychological resilience; IMFPE, intrinsic motivation for physical exercise. ***P < 0.001; **P < 0.01.

The bias-corrected bootstrap method was employed with 5,000 resampling iterations to estimate the mediating effects of each pathway and their 95% confidence intervals. The results are presented in Table 4. The total effect of the model was −0.120 (Boot SE = 0.038, 95% CI = [−0.045, −0.120]). The direct effect was −0.045 (Boot SE = 0.035, 95% CI = [0.023, −0.045]), with the confidence interval including zero, indicating that after including the mediating variables, the direct predictive effect of T1 ACEs on T2 intrinsic motivation for physical exercise was not significant. The total indirect effect was −0.075 (Boot SE = 0.020, 95% CI = [−0.116, −0.038]), with the confidence interval excluding zero, accounting for 62.45% of the total effect.

**TABLE 4:** The direct and indirect effect of chain mediation model.

Effect types	Intermediary path	Effect	Boot SE	Relative mediation effect	95% CI
Total effect	T1 ACEs→T2 MIL→T2 IMFPE	−0.120	0.038	100%	[−0.045, −0.120]
Direct effect		−0.045	0.035	–	[0.023, −0.045]
Total indirect effect		−0.075	0.020	62.53%	[−0.116, 0.038]
Mediating effect 1		−0.021	0.010	17.15%	[−0.044, −0.004]
Mediating effect 2	T1 ACEs→T2 PR→T2 IMFPE	−0.032	0.012	26.31%	[−0.058, −0.009]
Mediating effect 3	T1 ACEs→T2 MIL→T2 PR→T2 IMFPE	−0.023	0.009	19.07%	[−0.044, −0.008]

ACEs, adverse childhood experiences; MIL, meaning in life; PR, psychological resilience; IMFPE, intrinsic motivation for physical exercise.

Among the three specific mediating pathways, Pathway 1 (T1 ACEs → T2 meaning in life → T2 intrinsic motivation for physical exercise) had an effect value of −0.021, with a 95% confidence interval of [−0.044, −0.004], excluding zero, and accounted for 17.15% of the total effect. Pathway 2 (T1 ACEs → T2 psychological resilience → T2 intrinsic motivation for physical exercise) had an effect value of −0.032, with a 95% confidence interval of [−0.058, −0.009], excluding zero, and accounted for 26.31% of the total effect. Pathway 3, the chain mediating pathway (T1 ACEs → T2 meaning in life → T2 psychological resilience → T2 intrinsic motivation for physical exercise), had an effect value of −0.023, with a 95% confidence interval of [−0.044, −0.008], excluding zero, and accounted for 19.07% of the total effect. The 95% confidence intervals for all three mediating pathways excluded zero, indicating that both the individual mediating effects of T2 meaning in life and T2 psychological resilience, as well as their chain mediating effect, in the relationship between T1 ACEs and T2 intrinsic motivation for physical exercise were statistically significant.

## 4 Discussion

### 4.1 The longitudinal association between adverse childhood experiences and college students’ intrinsic motivation for physical exercise

The present results indicate that early adverse childhood experiences were significantly negatively associated with college students’ intrinsic motivation for physical exercise measured 6 months later, supporting Hypothesis H1. This finding provides longitudinal empirical evidence for understanding individual differences in exercise motivation from an early developmental perspective. Although studies directly examining the longitudinal relationship between ACEs and exercise intrinsic motivation remain scarce, substantial evidence has demonstrated that early adversity constitutes an important distal risk factor for various adverse health behaviors and physical and mental health problems in adulthood (Al-Shoaibi et al., 2024; Harada et al., 2021; Karatekin and Hill, 2021; Schmidt et al., 2025). These findings are consistent with the logic of the Cumulative Risk Model, namely, that greater exposure to early adversity is associated with an increased likelihood of health behavior risks in adulthood (Bertele et al., 2022; Nurius et al., 2015).

One possible explanatory pathway for this longitudinal association is that early adverse experiences, such as abuse and neglect, may fundamentally impair the fulfillment of individuals’ basic psychological needs (Liu et al., 2025b). According to Self-Determination Theory, when the needs for autonomy, competence, and relatedness are chronically frustrated during development, it becomes difficult for individuals to establish a motivational system rooted in intrinsic interest and personal values (Deci and Ryan, 2000). Individuals raised in abusive or neglectful environments may either avoid challenging physical activities due to a low sense of competence or withdraw from group-based physical activities due to difficulties in social interaction (Viveiros et al., 2025). This early-formed deficiency in exercise motivation may persist into early adulthood, manifesting as a lack of enduring internal interest in physical exercise (Nazligül et al., 2023). Research on positive parenting environments indirectly supports this explanation, showing that parental supportive behaviors can effectively enhance adolescents’ exercise motivation and adherence by satisfying their basic psychological needs (Meerits et al., 2023; Zeng et al., 2022). Taken together, evidence from both positive and negative perspectives indicates that the quality of the early developmental environment may profoundly influence individuals’ subsequent motivation to engage in physical exercise through pathways that either support or hinder the fulfillment of basic psychological needs. It is worth noting that ACEs explained only 1.4% of the variance in exercise motivation, suggesting that while early adversity does have an influence, it is not deterministic. This highlights the need to attend to other proximal psychological resources–such as meaning in life and psychological resilience–that may be more directly targeted in intervention efforts. Nevertheless, from a life-course perspective, students’ early developmental backgrounds remain an important contextual factor to consider when designing campus-based physical health promotion programs, as they may shape the very psychological resources that drive exercise motivation.

### 4.2 The mediating role of meaning in life

This study found that meaning in life played a mediating role between adverse childhood experiences and college students’ intrinsic motivation for physical exercise, with a mediating effect value of −0.021, thus supporting Hypothesis H2. The results indicate that early adversity does not directly diminish exercise motivation; rather, it exerts a negative influence indirectly by depleting individuals’ meaning in life (Zhao et al., 2025). This finding aligns with the theory of cumulative advantage or disadvantage, which suggests that the negative effects of early adversity may progressively erode meaning in life, and these deleterious effects accumulate over the life course, ultimately manifesting as attenuated motivation for specific health behaviors (Easton and Kong, 2021; Shi and Wu, 2020).

Hopelessness theory provides an explanatory framework for understanding this mechanism (Metalsky et al., 1993). Individuals who have experienced childhood trauma may develop feelings of helplessness and hopelessness due to the perception that past negative events are unchangeable (Liao et al., 2025). Such negative cognitive tendencies can impede the active construction of life goals, meaning coherence, and self-worth, leading to a diminished sense of meaning in life (Fan et al., 2024; McCormack and Thomson, 2017). Prior research has shown that individuals who experience chronic social exclusion report lower levels of meaning in life (Stillman et al., 2009). Concurrently, a positive association exists between meaning in life and motivation to engage in physical exercise (Sutin et al., 2021, 2022). Individuals with a stronger sense of meaning in life typically possess clearer life direction, higher self-efficacy, and richer positive emotional experiences. This makes physical exercise more likely to be perceived as valuable, enjoyable, and congruent with self-actualization, thereby generating a stronger intrinsic drive to participate. In contrast, individuals lacking meaning in life may lack the psychological energy and autonomous will necessary to explore and persist in effortful activities, tending instead to view exercise as an uninteresting burden (Pedro et al., 2012; Rodrigues et al., 2023). Taken together, early traumatic experiences may first lead to a loss of meaning in life, and this loss is further associated with reduced intrinsic motivation for physical exercise among college students. These findings underscore the important role of meaning in life as a core psychological resource that may link early adversity to subsequent health behaviors. At a practical level, this suggests that universities could help students with a history of childhood trauma reconstruct meaning in life, identify life goals and value directions, and gradually internalize physical exercise as an important pathway for self-actualization through life education curricula and career planning guidance.

### 4.3 The mediating role of psychological resilience

This study found that psychological resilience played a significant mediating role between adverse childhood experiences and college students’ intrinsic motivation for physical exercise, with a mediating effect value of −0.032, thus supporting Hypothesis H3. This result indicates that a greater number of early adversity experiences was associated with lower levels of psychological resilience, which in turn contributed to weaker intrinsic drive to engage in physical exercise (Morgan et al., 2021; Tang et al., 2025). This finding is consistent with prior research and further supports the view that psychological resilience serves as a mediator between adverse environments and positive behaviors (Nishimi et al., 2021).

Psychological resilience is a multidimensional positive psychological trait encompassing tenacity, optimism, and goal orientation. It can help individuals maintain or restore good psychological adaptation when confronting stress and adversity. As a chronic early stressor, adverse childhood experiences may continuously deplete individuals’ internal psychological resources, leaving them with relatively scarce reserves of psychological resilience when facing later life challenges (Shen et al., 2021; Yuan et al., 2024). Highly resilient individuals typically possess stronger emotion regulation abilities, more positive and flexible cognitive patterns, and more constructive coping strategies (Liu et al., 2024). These characteristics enable them to more effectively overcome inertia and obstacles when engaging in activities such as physical exercise that require sustained effort, and to maintain a relatively stable intention to participate (Hu et al., 2025; Tang et al., 2025; Zhang et al., 2025). In contrast, individuals with lower levels of psychological resilience may be more prone to giving up when encountering fatigue, setbacks, or external distractions during exercise, and their intrinsic motivation for physical exercise may therefore be difficult to sustain. The present data provide empirical support for this pathway, suggesting that psychological resilience may constitute an important psychological pathway linking early adversity to college students’ exercise intrinsic motivation. In terms of practical intervention, university physical education instructors and mental health educators could design structured adversity coping training, stress management courses, and progressive exercise challenge programs, allowing students with a history of trauma to gradually accumulate successful experiences in controlled setback situations, thereby enhancing their willingness to persist and their confidence in coping when facing physical activity challenges.

### 4.4 The chain mediating effect of meaning in life and psychological resilience

These results also indicate that meaning in life and psychological resilience may form a significant chain mediating pathway in the longitudinal association between adverse childhood experiences and college students’ intrinsic motivation for physical exercise, with a mediating effect value of −0.023, and the direct effect was attenuated to non-significance after the mediators were included, thus supporting Hypothesis H4. Specifically, the indirect effect of the chain pathway involving meaning in life and psychological resilience was significant, whereas the direct effect of adverse childhood experiences on college students’ intrinsic motivation for physical exercise became non-significant after the two mediating variables were included.

We first examine the mediating effect of meaning in life on psychological resilience. Although both meaning in life and psychological resilience fall within the domain of positive psychological capital, they may differ in the level of psychological functioning they entail. Meaning in life, as an individual’s fundamental cognitive awareness of the purpose and value of their existence, constitutes a more foundational meaning-making system. It provides individuals with a cognitive framework for understanding the world, interpreting experiences, and planning for the future. When this foundational resource is damaged by early trauma, it becomes difficult for individuals to extract a coherent life narrative from past negative experiences and to form positive expectations for the future (Akdag et al., 2024; Ostafin and Proulx, 2020). Psychological resilience, on the other hand, is more directly manifested in individuals’ capacity for adjustment and recovery when facing specific stressful situations (Troy et al., 2023); one of the prerequisites for its effective functioning is that individuals possess a relatively stable value and belief system as internal support (Sisto et al., 2019). In other words, when individuals lack a clear perception of “why to live” or “why to persist,” their ability to cope with adversity may be correspondingly weakened (Rose et al., 2023). Therefore, the erosion of psychological resilience by early adversity is partially achieved indirectly through the prior weakening of individuals’ meaning in life.

We next interpret the attenuated direct effect observed in this model. The non-significant direct effect of adverse childhood experiences on college students’ intrinsic motivation for physical exercise indicates that within this specific model, the association is entirely transmitted indirectly through the chain process of meaning in life and psychological resilience. This result suggests that early traumatic experiences do not, in themselves, absolutely determine the deficiency in college students’ motivation for physical exercise. If these two modifiable psychological variables–meaning in life and psychological resilience–can be effectively restored and cultivated during the critical period after trauma has occurred but before motivation has declined, there is hope of disrupting the negative influence of early adversity on the development of adverse health behaviors (Chen et al., 2021; Higgen et al., 2022; Howell et al., 2021). In this sense, the attenuation of the direct effect does not imply that the influence of early adversity has been fully explained; rather, it reveals the high plasticity of the pathways through which it is transmitted, providing both a theoretical rationale and an empirical basis for developing targeted psychological interventions in university settings.

Of particular note, among the three indirect pathways, the mediating effect of the single pathway through psychological resilience (26.31%) was the largest, exceeding that of the single pathway through meaning in life (17.15%) and the chain mediating pathway combining both (19.07%). This suggests that psychological resilience represents a particularly critical mediating variable linking adverse childhood experiences to intrinsic motivation for physical exercise. The predominance of psychological resilience among the three pathways may be attributable to its more direct role in individuals’ coping processes when confronting specific behavioral challenges. Although meaning in life provides a more foundational value and belief system, its influence on exercise motivation often requires longer-term cognitive integration (Hooker et al., 2024); in contrast, psychological resilience is directly involved in the immediate psychological processes of overcoming inertia and regulating negative emotions during each exercise decision, and thus has a closer functional connection with the initiation and execution of exercise behavior (Wang et al., 2025). This may be because psychological resilience operates as a proximal volitional resource that directly facilitates behavioral initiation and persistence in the face of challenges, whereas meaning in life serves as a more distal cognitive resource that requires longer-term integration to influence specific behaviors (Wang et al., 2025). This finding suggests that, in university intervention practices with limited resources, enhancing psychological resilience may represent a more rapid, effective, and impactful pathway (Peng et al., 2025). This is not, of course, to negate the role of meaning in life, but rather to emphasize the unique and central position of psychological resilience in the intervention chain–the enhancement of meaning in life ultimately needs to be translated into stronger psychological resilience in order to influence specific health behaviors more fully (Li D. et al., 2025).

### 4.5 Implications and limitations

This study offers several implications for the integrated practice of physical education and mental health services in university settings. First, attention should be directed toward high-risk yet often overlooked student groups. Screening for early adverse experiences should be appropriately incorporated into university mental health services and physical health promotion efforts. For students with a history of childhood trauma, targeted support can be provided through group counseling or individual therapy to help restore the basic psychological needs impaired by trauma, thereby creating conditions for rebuilding exercise motivation. Second, cultivating meaning in life represents an important direction for intervention. Integrating meaning education into course instruction, campus activities, and career guidance can help students explore personal values and establish life goals, thereby transforming positive perceptions of meaning in life into an intrinsic drive to engage in health-promoting behaviors, such as physical exercise. Third, the systematic cultivation of psychological resilience should not be overlooked. Through adversity cognition education, stress management training, and the development of positive psychological qualities, students’ capacity to cope with challenges can be effectively enhanced, buffering the negative impact of early or current stressful events, maintaining the relative stability of the motivational system, and providing psychological support for sustained participation in physical exercise. Given that resilience accounted for the largest mediating effect, resilience-oriented interventions may be more effective than generic motivation-based strategies in trauma-informed sport contexts. Fourth, the campus exercise environment should be optimized. Physical education instructors and coaches should actively adopt autonomy-supportive teaching approaches and create a safe, encouraging, and enjoyable exercise atmosphere to meet students’ needs for competence, relatedness, and autonomy, thereby facilitating the transition from controlled to autonomous exercise motivation.

This study also has several limitations. First, regarding the research design, although a two-wave longitudinal design was employed, both mediators and the dependent variable were measured concurrently at T2, which limits our ability to draw strong causal inferences about the temporal ordering among the mediators and between the mediators and the outcome. Future research should adopt three-wave or multi-wave longitudinal designs with temporally separated assessments of mediators and outcomes to more rigorously test the directionality of these relationships. Second, the sample was drawn from only two universities in Hunan Province using convenience sampling, which may limit the generalizability of the findings to broader populations. Although Hunan Province is geographically representative as a central region bridging eastern and western China, future studies should expand to multiple provinces and institutions with more diverse student populations and employ probability sampling methods to enhance external validity. Third, all measures were based on participants’ self-reports, which are susceptible to recall bias and social desirability. In particular, the retrospective measurement of ACEs may be influenced by current mood states–such as depressive symptoms–that could inflate or distort the reporting of past adverse experiences. Future research could incorporate prospective measures or multi-informant reports to supplement retrospective self-reports. Fourth, due to the anonymous nature of the survey and confidentiality requirements, we were unable to retain the identifying information of participants who completed only T1; thus, a formal attrition comparison between the final sample and dropouts could not be performed. Future studies should consider tracking participant characteristics at initial recruitment to enable attrition checks while maintaining confidentiality. Fifth, although the indirect pathways fully accounted for the total association in our specific model, we acknowledge that unmeasured confounding variables (e.g., social support, depression, family environment) may exist, and our model represents one plausible theoretical account rather than an exhaustive explanation of the mechanisms linking ACEs to exercise motivation.

## 5 Conclusion

Based on a longitudinal design, the present findings suggest the potential chain mediating role of meaning in life and psychological resilience in the longitudinal association between early adversity and college students’ intrinsic motivation for physical exercise. These findings may enrich theoretical understanding of the developmental origins of exercise intrinsic motivation and provide empirical evidence for university interventions aimed at promoting physical exercise through the cultivation of positive psychological qualities.

## Data Availability

The raw data supporting the conclusions of this article will be made available by the authors, without undue reservation.
